# Multimodal imaging of torpedo maculopathy in a Chinese woman: a case report

**DOI:** 10.1186/s12886-019-1161-7

**Published:** 2019-07-19

**Authors:** Yuhua Ding, Bangtao YAO, Hui Ye, Yan Yu

**Affiliations:** 1 Department of Ophthalmology, Jiangsu Province Hospital, The First Affiliated Hospital of Nanjing Medical University, Nanjing, Jiangsu Province, China; 2 Department of Ophthalmology, Lishui District People’s Hospital, Nanjing, Jiangsu Province, China

**Keywords:** Torpedo maculopathy, Multimodal imaging, RPE, OCTA

## Abstract

**Background:**

Torpedo maculopathy is a rare, benign, and congenital macular lesion that typically appears in a ‘torpedo-shape’ and is located at the temporal macula region. This study aimed to describe in detail regarding torpedo maculopathy in a Chinese woman using multimodal imaging.

**Case presentation:**

A 30-year-old Chinese woman with occasional yellowish-white macular lesions in her right eye during a routine examination was presented to our hospital. She had no other symptoms, and the best-corrected visual acuity of both eyes was 6/6. Funduscopic examination revealed a torpedo-shaped and mild hypopigmented lesion in the temporal macular area of her right eye. Infrared fundal (IR) images showed visible lesion contour, transverse elliptical, and with a tip pointing towards the central fovea of the macula. Microperimetry visual field appeared normal. The spectral-domain optical coherence tomography (SD-OCT) showed a normal inner retina, with mild thinner outer retina and retinal pigment epithelium in the temporal macular area, and correspondingly increased choroidal reflectivity. Other OCT findings included outer retinal loss/attenuation with significant atrophy of an intact ellipsoid zone. OCT angiography (OCTA) of choroid capillary layer revealed increased density of choroidal vasculature in corresponding to the area of the lesion, while the superficial and deep layers revealed normal vasculature. Fundus autofluorescence (FAF) revealed normal signal with slight hyperautofluorescence at the nasal lesion margin. Fundus fluorescence angiography (FFA) of the lesion showed variegated fluorescence and no leakage and change in the morphology during the whole imaging process.

**Conclusions:**

This is the first report to include a thorough and detailed description of torpedo maculopathy by using fundal photograph, IR, microperimetry visual field, OCT, OCTA, FAF, and FFA. Multimodal imaging provides precious and detailed information to further clarify the characteristics and development of this rare disease.

## Background

Torpedo maculopathy is a rare, benign, well-defined and congenital retinal pigment epithelium (RPE) disease that typically appears as a ‘torpedo-shaped’ lesion in the temporal macula. It was first reported by Roseman and Gass in 1992 [1] as an asymptomatic, solitary hypopigmented nevus of the retinal pigment epithelium. In the classic fundus, it manifests as a solitary hypo-pigmented lesion that is oval in shape, resembles a ‘bullet’ or ‘torpedo,’ with a wedge-shaped tail extending outward and points towards the foveola along the horizontal raphe [2]. Torpedo maculopathy was named by Daily in 1993 [3] due to its typical appearance. The typical fundus can be distinguished from other lesions, such as toxoplasma scar, traumatic injury, congenital hypertrophy of the RPE (CHRPE), and congenital RPE hypertrophia associated with Gardner syndrome, allowing for diagnosis.

However, the etiology of torpedo maculopathy remained unknown. Many imaging devices have been used to observe the lesion. We herein used multimodal imaging for describing torpedo maculopathy in a Chinese woman. To our knowledge, this is the first report in the world to observe torpedo maculopathy by using fundal photographs, IR, microperimetry visual field, optical coherence tomography (OCT), optical coherence tomography angiography (OCTA), fundus autofluorescence (FAF), and fundus fluorescence angiography (FFA). We hypothesized that this case might be in a very early stage or is a mild type of torpedo maculopathy.

## Case presentation

**Fig. 1:**
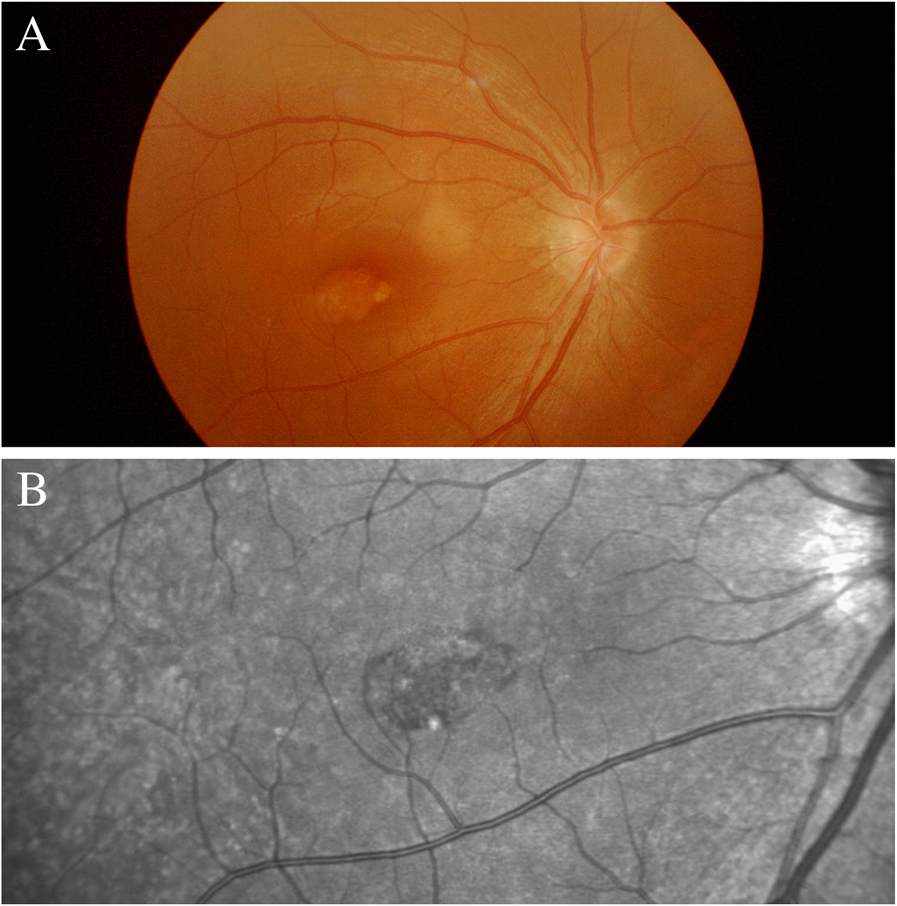
In the right eye, a spindle-shaped yellowish-white and hypo-pigmented lesion, which was about 0.5 disc diameter vertically by 1 disc diameter horizontally, was located in the temporal macular area with a tip pointed towards the central fovea of the macula (Fig. 1, A). IR photograph showed that the contour of the lesion was visible, and transverse elliptical and was consistent with the colorful fundus photograph (Fig. 1, B)

**Fig. 2:**
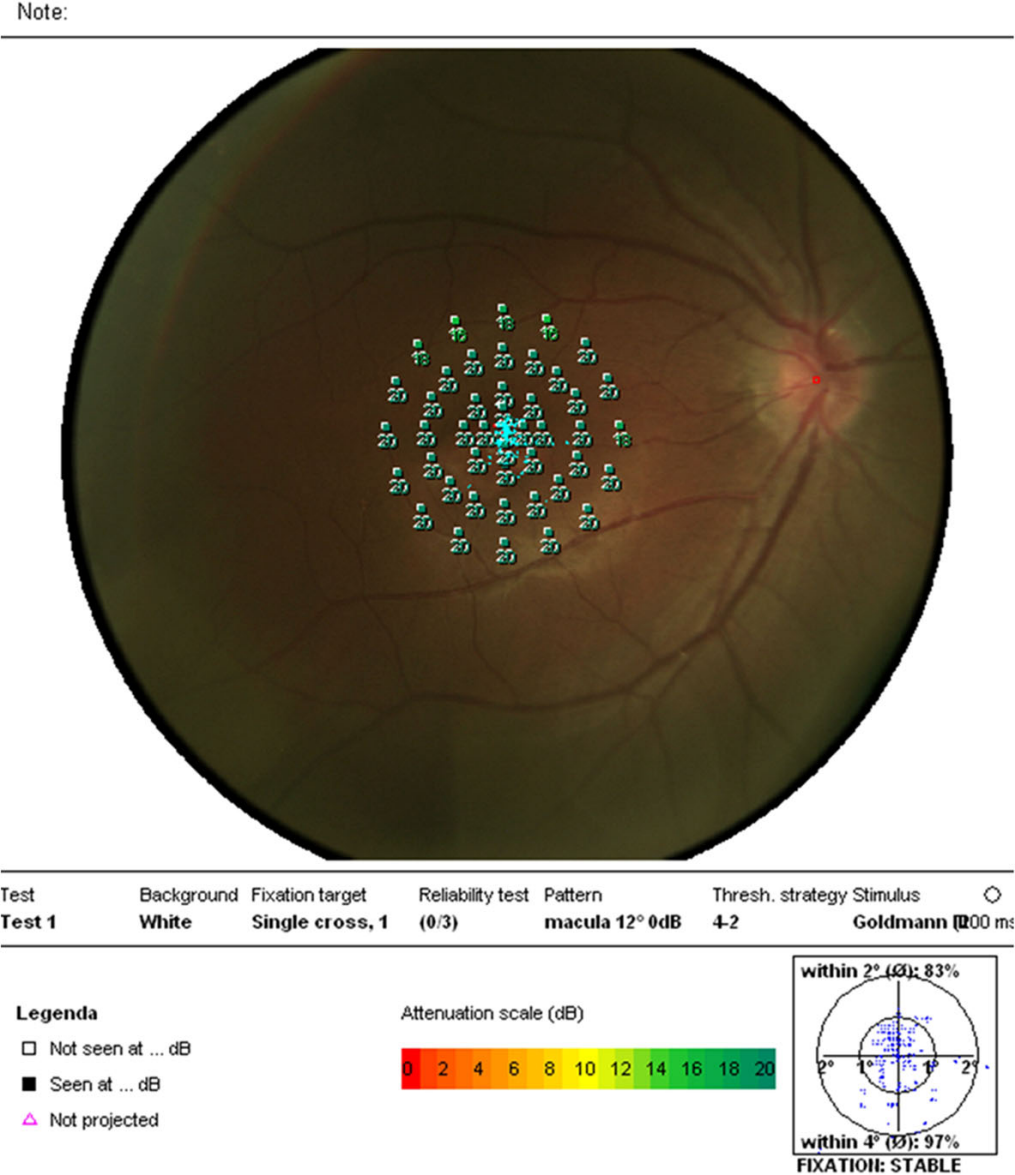
Microperimetry visual field was basically normal (Fig. 2)

**Fig. 3:**
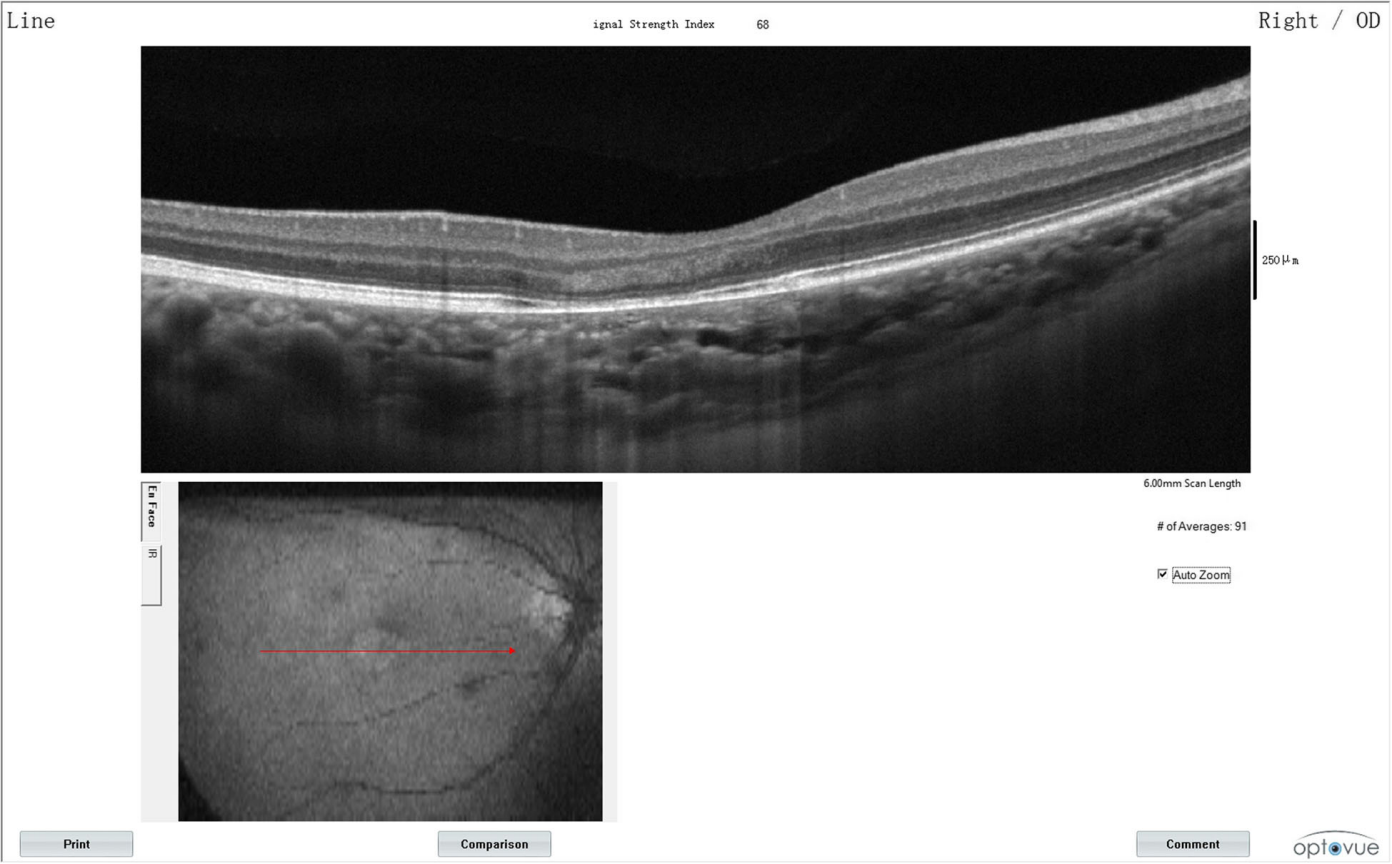
The SD-OCT showed a normal inner retina, mild thinner outer retina and RPE in the temporal macular area, with correspondingly increased choroidal reflectivity (Fig. 3)

**Fig. 4:**
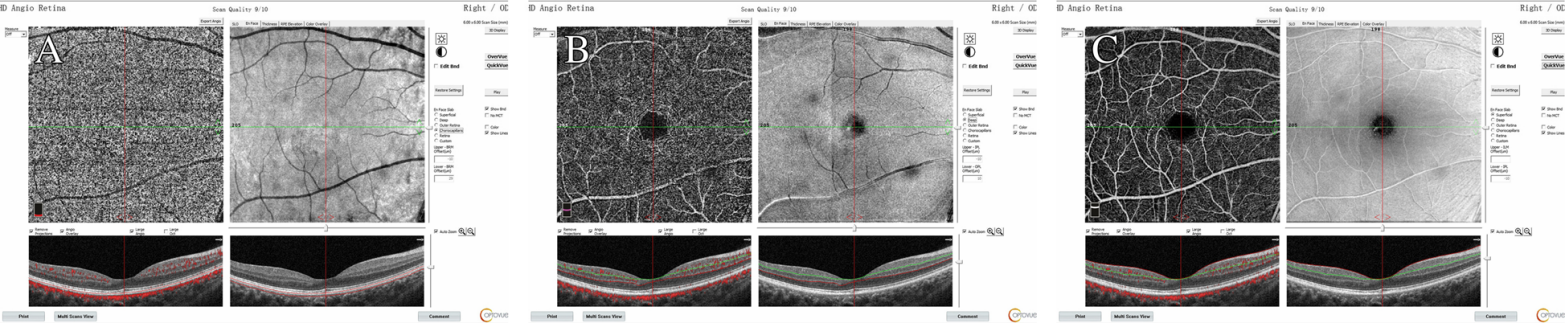
OCTA of choroid capillary segment revealed increased density of the choroidal vasculature, corresponding to the area of the lesion (Fig. 4, A). OCTA of deep retinal layer (Fig. 4, B) and superficial retinal layer (Fig. 4, C) appeared normal

**Fig. 5:**
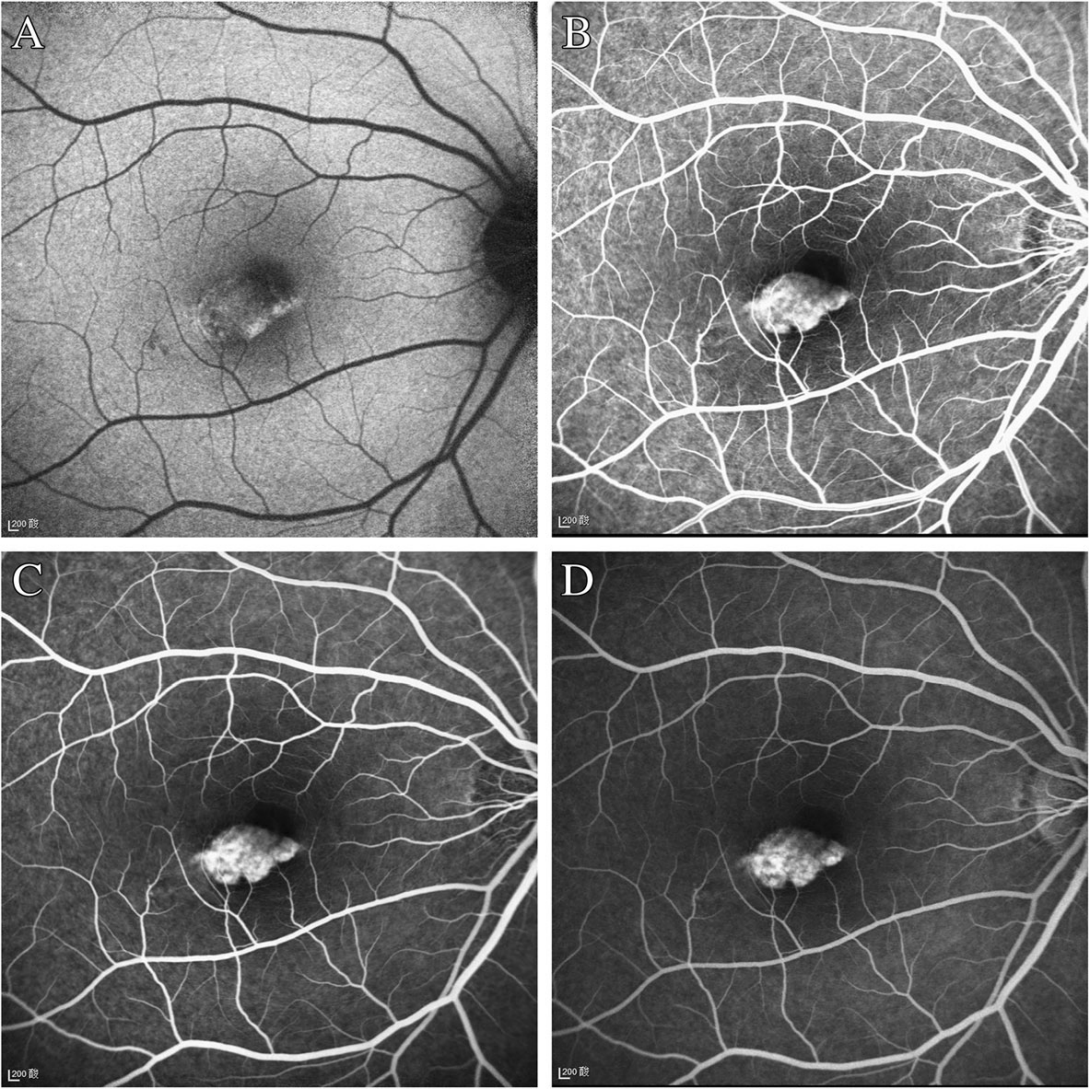
With FAF, the lesion showed normal signals mostly with slight hyperautofluorescence at the nasal lesion margin (Fig. 5, A). FFA of the lesion showed variegated fluorescence and no leakage and change in the morphology during the whole imaging process (Fig. 5, B,C,D)

A 30-year-old Chinese woman with yellowish-white macular lesion in right eye during a routine examination presented to our hospital. She had no other symptoms, and had no pain or vision loss in her right eye. The patient has no traumatic history. Her past medical history and ophthalmic history were negative. The initial best-corrected visual acuity (BCVA) was 6/6 for both eyes. The cornea was clear, and the anterior segment was normal. Pupils were equal, round and reactive to light with no afferent pupillary defect. There was no cataract in both eyes. The initial intraocular pressure (IOP) was 14 mmHg in the right eye and 13 mmHg in the left eye. Funduscopic examination of the left eye was unremarkable. A spindle-shaped yellowish-white and hypo-pigmented lesion of about 0.5 disc diameter vertically and by 1 disc diameter horizontally was located in the temporal macular area with a tip pointing towards the central fovea of the macula (Fig. 1, A). The IR photograph showed that the contour of the lesion was visible, and transverse elliptical and was consistent with the colorful fundus photographs (Fig. 1, B). Microperimetry visual field was basically normal (Fig. 2). The SD-OCT showed a normal inner retina, mild thinner outer retina and RPE in the temporal macular area, with correspondingly increased choroidal reflectivity (Fig. 3). Other OCT findings included outer retinal loss/attenuation with significant atrophy of an intact ellipsoid zone. OCTA of choroid capillary layer revealed increased density of the choroidal vasculature corresponding to the area of the lesion, while the superficial and deep layers appeared normal (Fig. 4). With FAF, the lesion showed normal signals mostly with slight hyperautofluorescence at the nasal lesion margin (Fig. 5, A). FFA of the lesion showed variegated fluorescence and no leakage and change in the morphology during the whole imaging process (Fig. 5, B,C,D). Based on these findings, the patient was clearly confirmed to have torpedo maculopathy.

The treatment for this patient was closely observed. The patient was followed-up every 3 to 6 months. Her BCVA remained still 6/6 in both eyes, and the fundus lesion still remained intact during follow-up at 15 months.

## Discussion

Torpedo maculopathy is a rare, benign, well-defined and congenital maculopathy, which is usually asymptomatic, but occasionally found during routine examination. It often occurs in patients without any relevant medical history and is most commonly unilateral, although bilateral cases have been reported previously [4]. The torpedo maculopathy appears as a transverse oval, yellowish-white hypo-pigmented lesion, and is located in the temporal macular area, with a tip pointing towards the central fovea.

Till now, the etiology of this disease still remained unclear. Few studies have been reported to explain the pathogenesis of this lesion. Pian et al. [5] assumed that it might be a developmental defect within the nerve-fiber layer at the horizontal raphe. Shields [6] suggested a persistent defect in the development of RPE during the fetal temporal bulge, which might be the reason for the cause of the lesion. Golchet et al. [7] hypothesized that the lesion may be related to dysmorphia of the emissary canal of the long posterior ciliary artery and nerve.

Microperimetry precisely revealed the correlation of retinal sensitivity and fundus lesion, and also detected microscotoma within the central visual field that may not be detectable with other standard perimetry methods [8]. Published reports have demonstrated that scotoma is frequently associated with torpedo lesion [9–11]. Focal RPE atrophy results showed that reduced metabolites and oxygen supply for the inner retina, secondary choriocapillaris loss and photoreceptor degeneration are associated with the reduction of retinal sensitivity [12]. This case was different from the earlier reports as there was no microscotoma seen, the retinal sensitivity appeared normal, and the patient had good visual function. Therefore, we speculated that the degree of RPE atrophy was mild in this case, and does not harm the function of the overlying photoreceptors.

Using SD-OCT, the outer retina was disorganized, and showed significant atrophy of RPE with an intact ellipsoid zone in this case. We thought that the RPE still preserved the function of ingesting photoreceptor cell outer segment, and so the ellipsoid zone still remained intact and the patient has normal visual function. Evan et al. [13] identified two patterns of abnormalities: type 1, attenuation of outer retinal structures without outer retinal cavitation; and type 2, those with both attenuation of outer retinal structures and outer retinal cavitation. According to their theory, this patient was included under type 1 torpedo maculopathy. Besides, they also observed that patients with type 1 lesion (age, 4–37) tended to be younger than those with type 2 lesion (age, 13–73) [13]. This patient was aged 30, and was consistent with the characteristics summarized by them.

OCTA non-invasively detects the movement of red blood cells to reveal the retinal and choroidal vascular system. Therefore, alterations in choriocapillaris can be visualized by using OCTA. Papastefanou and his colleagues [14] have described the features of torpedo lesions with OCTA, and revealed choroidal vascular segmentation with hypo-reflectivity (atrophy), which in turn showed correlation with the OCT of subretinal cleft. While there was no subretinal cleft observed in our patient, and the OCTA findings were different. OCTA choroid capillary segment revealed increased density of choroidal vasculature, revealing thinner RPE as optical signal transmission for increased choroidal thickness; however, the superficial and deep layers appeared normal. Comparison of our case with other previous reported cases [14] revealed early stage in our case according to the OCT classification.

Autofluorescence signal is predominantly derived from lipofuscin within the RPE [15]. In our case, FAF showed normal signals mostly with slight hyperautofluorescence at the nasal lesion. The possible explanation for this was due to the attenuation of both RPE and outer nuclear layer.

FFA of the lesion showed variegated fluorescence and no leakage and change in the morphology during the whole imaging process. This demonstrated no choroidal neovascularization (CNV), while few other cases showed the existence of CNV [16]. Lesions in our case were still limited to RPE without CNV.

Differential diagnosis such as posterior uveitis should be carefully addressed. Posterior uveitis is also known as choroiditis, which is ill-defined, and characterized by vitreous exudation and choroidal vasodilatation, resulting in CNV and visual reduction. OCTA, FFA and FAF examinations of posterior uveitis are distinguished from torpedo maculopathy. Early hypo-fluorescence followed by late leakage on FFA was observed in active choroiditis lesions. However, healed lesions presented hypo-fluorescence during the early phase on staining but no leakage was observed in the late phase. According to FAF, ill-defined hyper-autofluorescence was observed in the active choroiditis lesions, while rounded edges and hypo-autofluorescence within the lesion were shown in healed choroiditis [17]. These are quite different from torpedo maculopathy.

However, there are several limitations in this study. The sample size of the study was small, and indocyanine green angiography was not performed as it helps in better understanding of the differential diagnosis of this disease. Besides, no further follow-up examination is considered to be a limitation for this study.

## Conclusions

A case of torpedo maculopathy by using fundal photographs, IR, microperimetry visual field, OCT, OCTA, FAF, and FFA has been presented for the first time. it might be in a very early stage or is a mild type of torpedo maculopathy. The natural development of torpedo maculopathy still remained unclear, whether it develops very slowly or does not develop. Multimodal imaging provides precious and detailed information to easily diagnose and understand this rare disease. This is a very rare disease, and requires more case reports worldwide and longer follow-up time to further understand the etiology, characteristics and development of this lesion.

## Data Availability

The datasets used and/or analyzed during the current study are available from the corresponding author on reasonable request.

## Abbreviations

BCVA: Best-corrected visual acuity
CHRPE: Congenital hypertrophy of the RPE
CNV: Choroidal neovascularization
FAF: Fundus autofluorescence
FFA: Fundus fluorescence angiography
IOP: Initial intraocular pressure
IR: Infrared fundal
OCTA: OCT angiographic
RPE: Retinal pigment epithelium
SD-OCT: Spectral-domain optical coherence tomographic
